# Multicentric reticulohistiocytosis revealing breast cancer: Report of a case with dermoscopic, radiological and therapeutic aspects

**DOI:** 10.1111/ajd.13687

**Published:** 2021-08-16

**Authors:** Massimo Dell’Antonia, Laura Atzori, Luca Pilloni, Caterina Ferreli

**Affiliations:** ^1^ Dermatology Clinic Department of Medical Sciences and Public Health University of Cagliari Cagliari Italy; ^2^ Pathology Service Department of Medical Sciences and Public Health University of Cagliari Cagliari Italy

**Keywords:** multicentric reticulohistiocytosis, histiocytosis, histiocytic disorder, breast cancer, paraneoplastic syndrome, histiocytoma

Dear Editor,

Multicentric reticulohistiocytosis (MRH) is a rare non‐Langerhans cell histiocytosis of unknown aetiology, and approximately 300 cases are reported in English Literature. Underlying malignancies have been associated with up to 25% of cases; thus, it can be considered a paraneoplastic syndrome.^1^ We present here a case of a 48‐year‐old woman affected with MRH, which was the first sign of breast cancer, focussing on dermoscopic and radiological characterisation of this case. Treatment of MRH is often challenging, but in our patient, a complete regression was achieved after tumour excision without any specific therapy.

**Figure 1:**
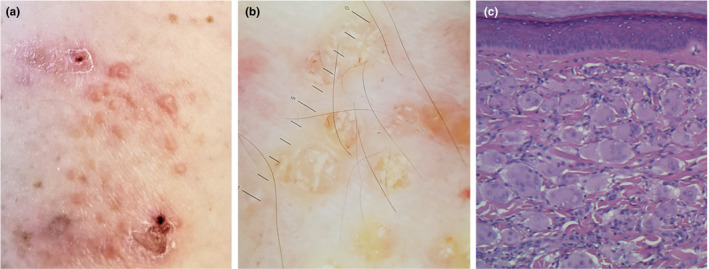
Multiple reddish orange‐yellow papules and scratching injuries of left forearm (a); dermoscopic examination shows yellow surface papules with chrysalis structures, white structureless areas and linear teleangectasias (b); histopathology of skin biopsy specimen shows giant multinucleated cells in dermis, haematoxylin and eosin original magnification ×40 (c)

A 48‐year‐old woman presented to our Department for a 10‐month history of multiple reddish orange‐yellow papules and nodules located on her hands, arms and head (Figure 1a). Dermoscopic examination of the lesions showed yellow surface papules with chrysalis structures, white structureless areas and linear teleangectasias (Figure 1b). The patient also had an 8‐month history of symmetrical inflammatory polyarthropathy involving the wrists, elbows, shoulders, knees, ankles and the distal interphalangeal (DIP) and proximal interphalangeal (PIP) joints of both hands. There was NSAIDs hypersensitivity in her medical history. A skin biopsy was performed, and histopathological examination showed the presence of histiocytic multinucleated giant cells with ground‐glass eosinophilic cytoplasm in the dermis (Figure 1c). A CD68 strain was diffusely positive, and the cells were negative for S100, CD1a and factor XIIIa. A diagnosis of MRH was made.

**Figure 2:**
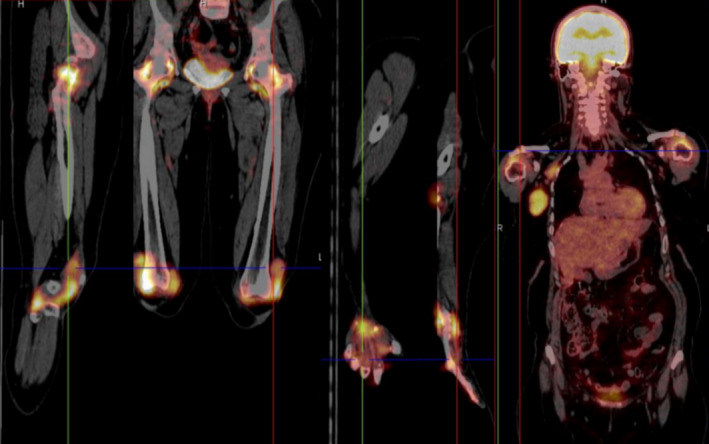
18FDG PET/CT image shows an abnormal uptake of FDG in DIP, PIP, wrists, shoulders, knees, right axillary lymph nodes and cutaneous lesions

Since MRH may be a paraneoplastic phenomenon, a 18F‐fluorodeoxyglucose positron emission tomography/computed tomography (FDG‐PET/CT) was performed to detect possible occult malignancies (Figure 2). 18FDG PET/CT showed an abnormal uptake of FDG in DIP, PIP, wrists, shoulders, knees and ankles. We considered these FDG uptakes as being secondary synovitis of these joints. Foci with increased FDG uptake were observed outside the DIP and PIP joints, corresponding the cutaneous papules and nodules. An elevated FDG uptake was also observed in right axillary lymph nodes, we considered this as a sing of possible internal malignancy, and we referred the patient to General Surgery Department for more investigations.

A lymph‐node biopsy and radiological investigations were performed, which detected a ductal carcinoma of the breast. According to Oncologists and General Surgeons, the patient was transferred to Oncology Department. We decided to treat MRH with alendronate 10mg DIE, since immunosuppressant and immunomodulator drugs were contraindicated, but the patient developed a drug eruption after 10 days of treatment, so we decided to stop the treatment and opted for a ‘wait‐and‐see’ approach.

The patient underwent mastectomy and axillary lymphadenectomy, followed by radiotherapy and tamoxifen therapy. We followed up the patient weekly and, surprisingly, the cutaneous lesion and the arthropathy improved from the third week after surgery and a complete regression was achieved in 3 months. To date, after 2 years of follow‐up, no relapse of MRH and breast cancer have been identified.

Multicentric reticulohistiocytosis (MRH) is a rare cell histiocytosis of unknown aetiology. The disease affects predominately skin and joints, but visceral involvement is possible.^1^

Skin involvement is characterised by multiple flesh‐coloured to reddish‐brown cutaneous papules and nodules, which mainly involve hands, face and arms.^1^ Dermoscopic examination could be useful in diagnosis: the detection of yellow/orange patches is highly suggestive of histiocytic infiltrate in dermis. Similar dermoscopic features have been described in granulomatous skin diseases, sarcoidosis and other histiocytic disorders,^2^ also characterised by granulomatous/histiocytic inflammatory infiltrate, which must be included in differential diagnosis.

Around nail folds can be found small papules with a characteristic coral‐bead appearance, and this feature is observed in about 30% of patients and represents a typical clinical sign of MRH.^3^

The diagnosis of MRH is performed on the basis of the histopathologic findings of cutaneous or synovial tissue specimens. The typical infiltrate is composed of histiocytes and multinucleated giant cells containing eosinophilic cytoplasm with a ground‐glass appearance.^4^

Radiological investigations should be performed in all patients with MRH to detect possible occult neoplasms. FDG‐PET/TC has high overall sensitivity and specificity for detecting cancer^5^ and has an important role in evaluation of inflammatory and granulomatous diseases since FDG is easy taken up by inflammatory cells^6^; thus, it is useful in evaluating the extent of the disease and assessing the possible association of neoplasms.

Treatment of MRH is challenging, and it generally consisted of immunosuppressant and immunomodulators, often contraindicated in patient with a malignant neoplasm. Bisphosphonates have proved effective on both skin and joint involvement, and they can be used as monotherapy or as combination therapy.^4^ In our case, a complete regression of MRH has been obtained after tumour excision, without any specific therapy, and this suggests that a ‘wait‐and‐see’ approach can be considered in patient with paraneoplastic MRH.
